# Disseminated Tuberculosis Mimicking Ankylosing Spondylitis

**DOI:** 10.1155/2011/195085

**Published:** 2011-09-06

**Authors:** Valérie Huyge, Serge Goldman, Muhammad S. Soyfoo

**Affiliations:** ^1^Department of Nuclear Medicine, Hôpital Erasme, Université Libre de Bruxelles (ULB), 1070 Brussels, Belgium; ^2^Department of Rheumatology, Hôpital Erasme, Université Libre de Bruxelles (ULB), 1070 Brussels, Belgium

## Abstract

Ankylosing spondylitis is a chronic inflammatory disorder affecting mainly the axial skeleton. Here we report a case of a man with a clinical suspicion of ankylosing spondylitis but with a persistence of increased inflammatory markers. In this case, ^18^F-FDG-PET/CT revealed multiple hypermetabolic lesions in axial skeleton, lymph nodes, and the lung, suggestive of either disseminated tuberculosis or lymphoma. Histological analysis of the pulmonary lesion revealed mycobacterium tuberculosis. This case highlights, firstly, the importance of excluding other diagnoses in the presence of clinical picture of ankylosing spondylitis and high inflammatory markers and, secondly, the determining role of PET/CT.

## 1. Introduction

Ankylosing spondylitis (AS) is a chronic inflammatory disorder affecting mainly the axial skeleton, the peripheral joints, and the entheses. The prevalence of AS is reported to be between 0.1 and 0.8% with more males being affected than females, in a 3 to 1 ratio [1]. AS is often diagnosed between the age of 20 and 40 years. The clinical spectrum of AS ranges from low back pain to systemic features including uveitis and renal failure [1]. More frequently, the symptoms of AS encompass inflammatory back pain, joint stiffness, and loss of spinal mobility. The diagnosis of AS is made according to the new ASAS criteria which include clinical, biological, and imaging features [2]. As such, patients suffering from AS might have increased inflammatory markers such as elevated C-reactive protein (CRP) levels and erythrocyte sedimentation rate (ESR) [3]. However, in patients with very high CRP and ESR, other causes should be searched for such as inflammatory bowel diseases [4]. Disseminated tuberculosis (DT) with insidious clinical presentation can mimic several clinical syndromes [5]. Bone involvement in DT is protean harvesting Pott's disease and Poncet's arthritis. Sacroiliac joint involvement in DT is rare (less than 10%) and reported as mainly unilateral [6, 7]. We hereby report the case of a young man with typical clinical features of AS, marked inflammatory syndrome, and bilateral sacroiliitis revealing DT.

## 2. Case Presentation

**Figure 1:**
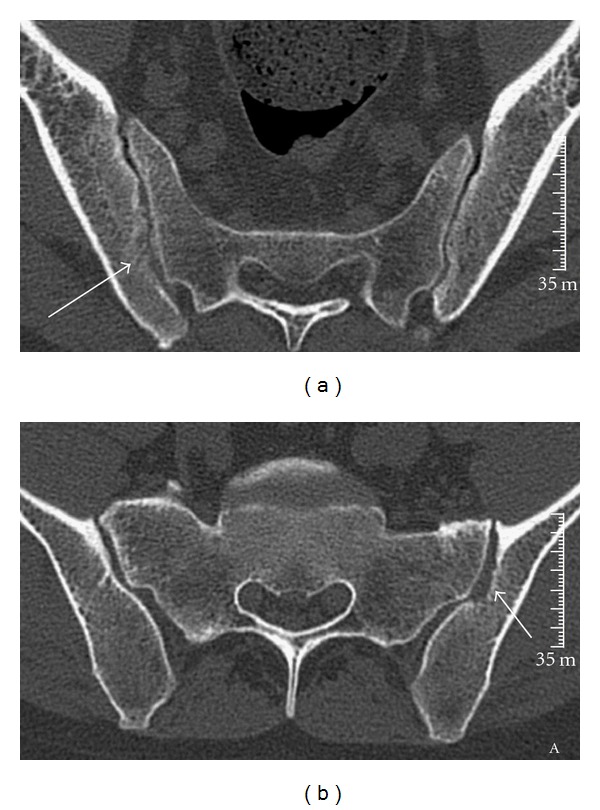
(a) CT scan demonstrates erosions on the right SIJ (*arrow*). (b) CT scan demonstrates erosions on the left SIJ (*arrow*).

A 30-year-old man, of East African origin, was admitted to our hospital for a constrictive chest pain. The patient's history included acute methanol intoxication with bilateral permanent blindness, due to necrosis in bilateral putamen demonstrated on magnetic resonance imaging (MRI). The patient was followed in the rheumatology outpatient clinic for AS. There was no history of smoking or illicit drug consumption but the patient admitted drinking alcohol occasionally. The patient reported low inflammatory back pain for more than one year, good response to nonsteroid anti-inflammatory drugs (NSAIDs) and computer tomography imaging (CT) of bilateral sacroiliitis (grade 2), leading to the diagnosis of AS according to the ASAS criteria (Figure 1).

The initial physical examination revealed a satisfactory general condition, no fever, normal vital signs, and synovitis of the knees. The cardiopulmonary examination was normal. Initial blood examination showed increased ESR at 80 mm/h (*N* < 20 mm/h) and CRP 5.8 mg/dL (*N* < 0.5 mg/dL), moderate hepatic cytolysis (<2X Normal values), and anicteric cholestasis. Total bilirubin, hematological tests, and renal function were normal. Troponin level was initially increased to 0.091 mg/dL; it decreased after 3 hours to 0.023 mg/dL. Chest radiography revealed no abnormalities. An electrocardiogram revealed widespread concave S-T elevation and a P-R depression (D2 D3). Cardiac echography was normal. Myocardial infarction was ruled out, and the patient was treated with NSAIDs with good clinical response.

**Figure 2:**
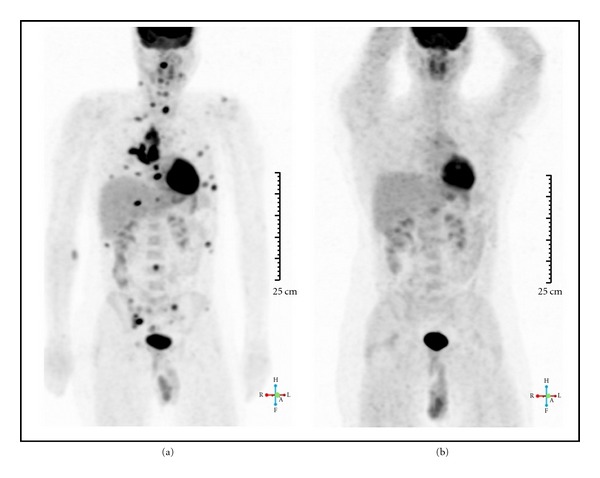
(a) represents ^18^F-FDG-PET/CT imaging showing multifocal hypermetabolic lesions of the sacroiliac joints, lungs and axillary, cervical, supraclavicular lymphadenopathy suggestive of systemic disease. (b) represents ^18^F-FDG-PET/CT imaging one year after antituberculosis treatment. All hypermetabolic lesions have been cleared, confirming efficient therapy of tuberculosis.

**Figure 3:**
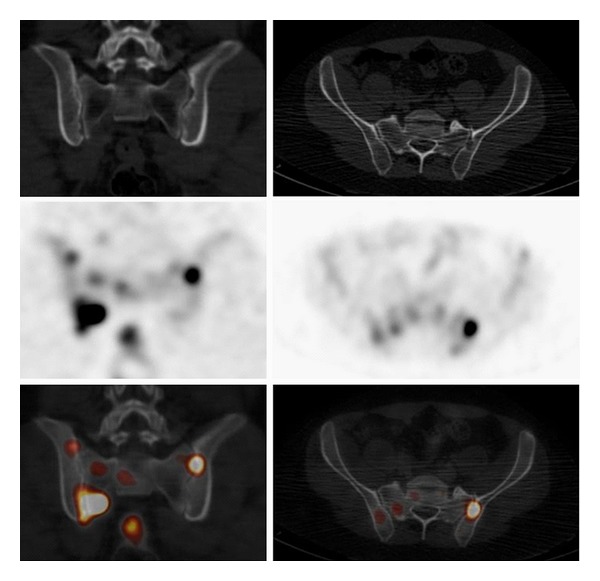
The figure shows sacroiliac joint involvement as hypermetabolic lesions as seen on ^18^F-FDG-PET/CT. Left row: coronal CT, PET, and fused PET/CT slices. Right row: transverse CT, PET, and fused PET/CT slices.

Because of the persistence of increased inflammatory markers and negative biological infectious investigations and first imaging studies (chest X-Ray and abdominal ultrasound), a whole-body positron emission tomography combined with a computed tomography (PET/CT) using F18-fluorodeoxyglucose as radiotracer (^18^F-FDG) was performed to detect any occult infectious or inflammatory sites. The ^18^F-FDG-PET/CT revealed mediastinal, hilar pulmonary, supraclavicular and cervical bilateral hypermetabolic lymph nodes, hypermetabolic condensation in the right lung, multiple hypermetabolic lesions of axial skeleton (Figure 2(a)), in particular the thoracic spine, rib cage, and bilateral sacroiliac joints (Figure 3). This was suggestive of either DT or lymphoma. A tuberculin skin test was done and was highly positive (22 × 27 mm), whereas analysis for TB was negative in the sputum and urine. Bronchoscopy with biopsy of the right pulmonary lesion was performed. Histological analysis of the lesions showed the presence of necrotic granuloma, and microbiological examination revealed mycobacterium tuberculosis. The diagnosis of DT with axial skeleton, lung, and lymph node involvement was retained, and the patient was treated with Isoniazid 300 mg/d, Rifampicin 300 mg bid, Pyrazinamide 500 mg/d, and Ethambutol 400 mg qid. ^18^FDG-PET/CT was repeated after 1 year of treatment and showed total disappearance of the aforementioned lesions (Figure 2(b)). 

## 3. Discussion

Skeletal lesions are present in 2% of all cases of tuberculosis, and sacroiliac joint (SIJ) involvement has been reported in up to 9.7% of patients with skeletal tuberculosis [5]. We here report the case of a patient presenting with clinical features of AS revealing multifocal skeletal tuberculosis with bilateral SIJ involvement. Pouchot and colleagues reported 11 cases of SIJ tuberculosis, but none of them had bilateral involvement [6]. Ramlakan and Govender reported 17 patients of SIJ tuberculosis, with only one case with bilateral involvement [7]. Multifocal skeletal tuberculosis is often difficult to diagnose because of the insidious presentation and the paucity of systemic symptoms such as fever, weight lost, and night sweat. Hong and colleagues also reported a case of multifocal skeletal tuberculosis, which was misdiagnosed as AS and did not respond to antirheumatic drugs [8]. Our patient was also initially diagnosed as AS because of clinical and bilateral sacroiliitis CT scan features but persisting inflammatory markers in our patient prompted further investigations. Indeed, patients with AS might present with increased inflammatory markers but those generally are normalized with NSAIDs [3]. Moreover, many patients with axial involvement have normal or slightly elevated values of ESR and CRP, in comparison to patients with predominant involvement of the peripheral joints, IBD, or rheumatoid arthritis [4]. A bone scan or an MRI of SIJ was not being performed because CT scan of SIJ already demonstrated bilateral sacroiliitis. Conventional imaging investigations were negative. A CT thorax and CT abdomen were not performed because of the high-dose irradiation of this examination and the possibility of access to a whole-body FDG-PET/CT low dose. This hybrid imaging technique is well accepted for routine assessment of oncology disorders [9] but it is also increasingly used for the assessment and monitoring of infectious and inflammatory processes [10–12]. Indeed, ^18^F-FDG accumulates not only in malignant tissues but also at the sites of infection and inflammation. Moreover, ^18^F-FDG-PET/CT is a whole-body procedure allowing delineation of both the localization and the number of foci, even at sites that are clinically silent. In the present case, polyarticular pain with bilateral sacroiliitis was the most salient clinical features inveigling the diagnosis of AS. Value of ^18^F-FDG-PET/CT in the diagnosis and assessment of multisystemic diseases is illustrated in this case with the detection of a lung lesion, lymphadenopathies, and bone lesions, including lesions of sacroiliac joints. As here shown, ^18^F-FDG-PET/CT also permits to guide biopsy procedures for pathological and bacteriological diagnosis, and it helps in the monitoring of response to therapy. 

This case highlights the importance of excluding other diagnoses in the presence of clinical picture of AS and high inflammatory markers. As such, ^18^FDG-PET/CT might be a valuable tool in the detection of inflammatory and infectious processes.
